# Perspectives on Physical Activity and Learning from Children With and Without ADHD

**DOI:** 10.3390/sports13080240

**Published:** 2025-07-22

**Authors:** Beverly-Ann Hoy, Maya Connolly Steinberg, Barbara Fenesi

**Affiliations:** Faculty of Education, Western University, London, ON N6G 1G7, Canada; bhoy6@uwo.ca (B.-A.H.); mstein49@uwo.ca (M.C.S.)

**Keywords:** ADHD in children, physical activity, child perspectives, classroom-based physical activity intervention, hyperactivity, physical activity participation, attention, mood

## Abstract

(1) Background: Children with attention-deficit hyperactivity disorder (ADHD) engage in significantly less physical activity than their peers. While ample research has shown the beneficial effect of physical activity on ADHD management, we have little to no knowledge of how children with ADHD experience physical activity, which may ultimately undermine the utility of prescribed physical activity programming. This study compared experiences and perspectives of physical activity in school and non-school settings, between children with and without ADHD. (2) Methods: In this study, 23 children with ADHD and 24 children without ADHD participated in semi-structured interviews, sharing their views on physical activity in school and non-school settings. (3) Results: Inductive content analysis revealed that, compared to children without ADHD, children with ADHD reported lower physical activity levels, more often emphasized the benefits of movement for improving mood and focus during learning, viewed classroom-based desk cycling as a helpful tool to focus their attention, and expressed a desire to use desk cycling during classroom learning. (4) Conclusions: This study emphasizes key differences in the physical activity experiences and preferences between children with and without ADHD; it also offers insight into how classroom learning may be enhanced by offering optional physical activity outlets for children who identify as benefiting from movement during learning.

## 1. Introduction

Attention-deficit hyperactivity disorder (ADHD) is one of the most common neurodevelopmental disorders, comprising three primary symptoms often arising in early childhood, including inattention, impulsivity, and hyperactivity [1]. Underlying these deficits are impaired executive functions, which are top-down mental processes needed for various cognitive abilities, such as inhibition, working memory and attentional switching [2]. ADHD is also marked by dysfunctional emotion regulation, often contributing to delays in learning and development [3,4]. In educational settings, children with ADHD frequently struggle with impulse control and sustained attention, resulting in difficulties listening to teachers and absorbing classroom instructions [5]. In social settings, children with ADHD often experience peer rejection and bullying, which contributes to diminished self-esteem and confidence, and increased symptoms of anxiety and depression [6]. Furthermore, approximately 33–50% of children with ADHD experience comorbid psychological challenges, such as anxiety, depression and oppositional-defiant disorder, further exacerbating social strain and academic difficulties [6,7,8]. The complex and heterogeneous nature of ADHD has led to the development of various pharmacological and behavioural treatments, such as stimulant (i.e., methylphenidate) and non-stimulant pharmacology (i.e., antidepressants and alpha-2 agonists), psychoeducation [9], cognitive therapy, neurofeedback, dietary interventions [10], and brain stimulation [11,12,13]. These therapies have variable efficacy and adherence, particularly stimulant treatments, with many behavioural management treatments being costly in terms of time and resources [14].

Physical activity has received extensive attention in the last decade as a promising adjunct treatment to help ameliorate ADHD symptoms and supplement existing treatments [15,16,17]. In neurotypical child populations, physical activity has been shown to support structural and functional brain health [17], with more active children showing greater executive functioning, psycho-emotional well-being and social connection compared to less active children [16]. Children with ADHD have a unique neuroanatomy [18] that makes them even more responsive to the effects of physical activity compared to neurotypical children [17,19]. Physical activity activates the same catecholaminergic systems as stimulant medications that children with ADHD are often prescribed [20] and improves cerebral blood flow throughout the brain; this has particular ramifications for the prefrontal cortex (PFC), which is hypothesized to be under-aroused in ADHD (i.e., the hypofrontality hypothesis [21]). The PFC drives higher-order executive functions and facilitates emotion regulation, which are the core deficits that children with ADHD often experience. Indeed, physical activity has been shown to improve core ADHD symptoms in children (i.e., inattention, hyperactivity, impulsivity), while also addressing associated symptoms such as internalizing and externalizing difficulties, social struggles, and sleep disturbances [22,23,24,25]. There is also a positive association between physical activity and ADHD symptomology, such that children with ADHD who engage in more physical activity have greater cognitive and behavioural outcomes compared to children with ADHD who engage in less physical activity [25,26,27]. Further, the Cognitive Energetic Model offers a nuanced perspective on ADHD, emphasizing that performance deficits may stem from suboptimal energetic states rather than solely from cognitive impairments [28,29]. For instance, individuals with ADHD might perform comparably to their peers under stimulating conditions but struggle in monotonous settings due to under-activation. This understanding underscores the importance of considering both cognitive and energetic factors when assessing and treating ADHD. Furthermore, the model bridges human cognitive neuroscience with neurobiological studies in animals, facilitating an integrated approach to researching the underlying mechanisms of ADHD. Educational classrooms that integrate physical activity into instructional time have also been shown to improve several outcomes for children with ADHD, including academic performance, school behaviour, classroom engagement, motivation, and on-task behaviour [30,31]. Often referred to as “brain breaks” or “activity breaks”, incorporating as little as four minutes of physical movement into curriculum-based learning during class time can help focus student attention and improve a myriad of academic-related outcomes for children with and without exceptionalities. Despite traditional education systems, especially in Western society, children cannot sustain their attention for long durations in a classroom and certainly cannot do so while being required to remain stationary for long periods of time. Bodily movement is directly related to the brain’s ability to function [21,30], especially for those with ADHD [32]; incorporating movement into classroom time not only breaks up learning demands but also honours the inherent needs of the body and ultimately promotes academic learning.

Despite the well-documented benefits of physical activity for children with ADHD, they are significantly less active than their neurotypical peers and are less likely to meet the World Health Organization’s (WHO) recommendation of 60 min of daily moderate to vigorous physical activity for brain and body health [33,34,35,36]. Insufficient physical activity participation among children with ADHD is prevalent both inside and outside of school settings [36]. Not only do children with ADHD participate in fewer sports and extracurricular activities [33,34,35,36], they also engage less with school-based physical activities [36]. One of the most salient barriers to physical activity participation among children with ADHD is when physical activities are dependent on ability, as their preferences are more commonly derived from enjoyment [36,37]. Identifying the limiting and motivating factors that determine physical activity engagement both inside and outside school settings is essential for creating informed opportunities in which children with ADHD will actually engage.

Research investigating determinants of children’s physical activity behaviours have relied on parent or teacher reports, ignoring child-centric perspectives [38]. This is problematic as large discrepancies have been documented between parent and child reports in domains related to physical activity participation, as well as emotional and behavioural challenges [38,39]. No evidence yet exists that highlights child-centric perspectives on physical activity barriers and facilitators. Involving children in qualitative and quantitative research can enhance research quality and increase the likelihood that findings are translated into real-world practice [40,41]. At the broader community level, without the active involvement of children in designing physical activity programming, such programs may fail to address specific needs, leading to poor implementation and low participation rates, ultimately reducing their effectiveness [41]. At the school level, children who are actively involved in designing and implementing school-based health activities have been shown to be more motivated and engaged, experience positive health outcomes and social interactions, and contribute to positive changes in school culture and climate [41,42]. Overall, there is an important need to learn from children about their perspectives of physical activity and effective programming both inside and outside school settings.

The current study is grounded in knowledge-based and phenomenological theoretical frameworks. The knowledge-based framework emphasizes how an individual’s actions, reasoning and decision making are informed by accumulated knowledge. This approach integrates findings from developmental, educational, and sports psychology, to highlight how developmental factors, self-regulation, and expertise in physical activity participation lead to perceptions of and participation in physical activity [34]. The knowledge-based approach suggests that physical activity participation is influenced by environmental cues and personal experience. This framework is valuable for examining how individuals, specifically children with ADHD, develop differing levels of proficiency in physical activity as a function of environmental cues and personal experience. Furthermore, the phenomenological approach emphasizes that to understand the nature of a phenomenon, the lived experiences of individuals must be considered [43]. Phenomenological research examines the qualities of an experience via qualitative methods, such as interviews with those who have first-hand experience with the phenomenon of interest [43]. In essence, to be better informed about physical activity experiences in children with ADHD, the phenomenological approach suggests constructing a knowledge base from their lived experiences and using that to inform evidence-based programming.

Taken all together, the current study explored the physical activity experiences of children with and without ADHD. We aimed to understand the similarities and differences in neurotypical and neurodivergent children’s physical activity practices and preferences, knowledge of physical activity benefits, and views of physical activity during classroom learning. It was hypothesized that children with ADHD would have fewer experiences with physical activity, less knowledge of physical activity benefits, and would have more favourable views of physical activity during classroom learning compared to their non-ADHD peers.

## 2. Materials and Methods

### 2.1. Participants

A total of 47 children aged 8–12 were recruited for this study, 23 children without ADHD (mean age = 10.04, males = 16, females = 7) and 24 children with ADHD (mean age = 9.83, males = 15, females = 9), from London, Ontario (Canada). Participants were required to have a formal ADHD diagnosis by a family physician or psychologist, as indicated by parental report. Thematic saturation was employed to determine when to discontinue data collection [44]. Saturation was achieved when no additional new information, as pertaining to the specific research questions, was being discussed during participant interviews. Researchers engaged in saturation consultation throughout the data collection process. Participants were recruited through flyers posted at the Institution’s Mary J. Wright Child & Youth Development Clinic and through community centres throughout London Ontario. Flyers were also emailed out to a list of current clients (guardians and children) at the Development Clinic who previously indicated that they would be interested in participating in research studies. Participants were excluded if they were not fully literate or did not speak English, if they had any developmental and neurological exceptionalities above and beyond ADHD, if they were unable to participate in moderate-intensity physical activity, and if they were colour blind.

### 2.2. Procedure

Data for this study came from a larger study examining the role of physical activity during executive functioning in children with and without ADHD [21]. In this larger study, children with and without ADHD completed an inhibitory control task (Stroop) [45,46] while remaining stationary (stationary condition) and while desk cycling (movement condition). The previous study is described in detail in Hoy et al. [21]. Relevant to the current study, following participation in the larger experimental procedure, children were interviewed about their physical activity experiences, barriers and facilitators, along with their perceptions of using the desk cycle during attention-demanding tasks, especially in a classroom setting. Interview questions are provided in Table 1, along with how they map onto our four key research questions. Participants verbally responded to interview questions, and their responses were recorded on a voice recorder. At the end of the study, participants and their guardians were debriefed, and researchers answered any remaining questions.

### 2.3. Analysis

Audio recordings of interviews were transcribed verbatim using Trint, an online transcription service. The transcribed interviews and audio recordings were read over and listened to independently by two researchers to ensure transcription accuracy and to familiarize researchers with the themes within participant responses. The transcriptions were organized based on participant, question, and study group (ADHD vs. non-ADHD). A formal codebook was then created for each research question, which consisted of defined themes and the frequency of participant endorsements. Each theme in the codebook held a minimum of three participant endorsements. In the transcribed interviews, comments were made to identify each instance of an endorsed theme, and frequencies of endorsements were verified by a second researcher. This allowed transcriptions and endorsements to be verified throughout the study process. Exemplar endorsements of each theme were highlighted and used to illustrate each theme in the codebook. All qualitative analyses were carried out using Microsoft Excel. A post-positivist lens was applied to the qualitative analysis [47]. This acknowledges researcher bias and potential influences of personal experience and background knowledge given that researchers had a preconception towards valuing the role of physical activity in child physical and mental health [48]. Inductive content analysis was used to examine the transcribed audio-recorded interviews. Interviews were analysed to identify trends and patterns, with frequent participant endorsements categorized using explicit codes. Content categories emerged directly from the data. The analysis focused on identifying and reporting common themes from the interview transcripts. Chi-squared tests were conducted using SPSS (version 26) on the frequency of endorsements for each research question. The chi-squared tests assessed whether the frequency differences between the ADHD and non-ADHD group were statistically significant, highlighting meaningful differences in physical activity experiences and knowledge between the two groups. Frequency tables were created for each research question and each group. The frequency tables included the themes of each question, exemplar quotations illustrating each theme, the overall frequency of endorsements, and the frequency of endorsements made by males vs. females. For the gender analysis only, each participant’s endorsement was counted once per theme, regardless of how many endorsements were made. The tables were in order of most frequently endorsed themes to least frequently endorsed themes by group.

### 2.4. Trustworthiness

Multiple measures were implemented to ensure trustworthiness. Data triangulation was employed, involving three investigators who engaged in the full research process. This helped aid in credibility by confirming findings across multiple investigators and by minimizing research bias [49,50]. Dependability was promoted by creating explicit and repeatable methods through the use of recruitment scripts and interview questions. Detailed methodology and research processes were recorded throughout the research process.

## 3. Results

Table 2 provides the demographic characteristics for participants with and without ADHD and their guardians.

Findings for each research question are provided below. Themes are presented in order of frequency of occurrence. Differences in theme frequency, as determined by chi-squared tests, between the ADHD and non-ADHD groups are provided below each table. Representative quotations from participants are provided in Supplementary File S1.

**Research Question** **1:** **What physical activities do children with and without ADHD typically participate in?**

Table 3 provides a summary of the physical activities in which children from both groups participate.

In summary, both groups expressed a wide range of physical activities in which they engage. However, children with ADHD reported engaging in significantly fewer individual activities such as biking and swimming as well as team sports compared to children without ADHD. A higher proportion of females in the non-ADHD group endorsed participating in team sports and individual activities, compared to females in the ADHD group. In addition, the ADHD group indicated a stronger preference for non-structured activities, whereas the non-ADHD group had a stronger preference for school-based activities.

**Research Question** **2:** **How do children with and without ADHD perceive the benefits of physical activity based on their (1) knowledge and (2) personal experience?**

1. *Perceptions of Physical Activity Benefits Based on Personal Knowledge*

Table 4 provides a summary of the perceived benefits of physical activity based on knowledge.

Overall, the non-ADHD group provided more endorsements for Theme 1 (body benefits) and Theme 3 (overall health benefits), whereas the ADHD group had a larger variety of responses when discussing physical activity benefits. In addition, there were more children with ADHD who endorsed Theme 4 (athletic performance) and Theme 5 (mental processes), compared to children without ADHD.

2. *Perceptions of Physical Activity Benefits Based on Personal Experience*

Table 5 displays a summary of the perceived benefits of physical activity based on personal experience.

Taken together, participants with ADHD described how physical activity specifically helps to improve their state of mind and allows them to be more positive. In addition, females with ADHD had the greatest proportion of endorsements for personal growth as a byproduct of physical activity engagement, whereas females in the non-ADHD group did not discuss personal growth at all.

**Research Question** **3:** **How do children perceive the impact of physical activity on their (1) focus and (2) mood?**

1. *The Effects of Physical Activity on Focus*

Table 6 provides a summary of the perceived effects of physical activity on participants’ focus.

2. *The Effects of Physical Activity on Mood*

Table 7 displays the themes related to participants’ perceptions of physical activity’s effect on mood.

Overall, participants with ADHD indicated that physical activity helps both their focus and their mood more frequently than those without ADHD. Interestingly, more males than females in the ADHD group endorsed physical activity improving their mood, whereas in the non-ADHD group, this finding was reversed.

**Research Question** **4:** **What are children’s opinions on using desk cycles during (a) learning tasks and (b) in classroom environments?**

1. *Use of the Desk Cycle During Learning Tasks*

Table 8 summarizes the main themes identified by participants regarding the use of desk cycles during learning tasks.

In summary, more participants with ADHD viewed using the desk cycle during learning tasks to be helpful, compared to those without ADHD. More males in the ADHD group viewed the desk cycle as helpful compared to females, and in the non-ADHD group, this finding was reversed. Females in the ADHD group viewed the desk cycle as the most distracting out of all groups.

2. *Use of the Desk Cycle in Classroom Learning Environments*

Table 9 summarizes the main themes endorsed by participants regarding using desk cycles in classroom contexts.

Overall, there were more participants with ADHD who endorsed a desire to use the desk cycle in classroom learning environments compared to those without ADHD. Within the ADHD group, there were more males who endorsed a desire to use the desk cycle compared to females. In contrast, in the non-ADHD group, more females endorsed a desire to use the desk cycle than males.

## 4. Discussion

The current study explored the physical activity experiences of children with and without ADHD. The findings revealed that children with ADHD participate in less physical activity overall, are less aware of the benefits of physical activity, and tend to focus on athletic performance as the main benefit of physical activity. Furthermore, children with ADHD expressed physical activity supports their mood to a greater extent than their non-ADHD counterparts and noted a greater desire to use the desk cycle during learning tasks and in classroom environments compared to those without ADHD. There were also notable gender differences across themes; males more frequently endorsed participation in competitive or performance-oriented activities and highlighted physical benefits, such as strength and athletic improvements. Females, particularly those with ADHD, tended to participate more in school-based and non-structured physical activity and more often emphasized mental health benefits. Taken altogether, the findings from this study underscore key differences in perceptions of and experiences with physical activity between children with and without ADHD and highlight the importance of adopting gender-responsive approaches when promoting physical activity, especially for girls with ADHD, whose opportunities for engagement and motivations may differ significantly from those of their male peers. In the following, we will further discuss these themes and provide suggestions for future research and educational integration.

**Research Question** **1:** **What physical activities do children with and without ADHD typically participate in?**

Children with ADHD reported participating in physical activity significantly less often than their peers without ADHD, both in individual activities (i.e., biking, dancing, swimming) and team sports (i.e., soccer, hockey, basketball). These findings align with previous research showing reduced physical activity participation among children with ADHD compared to their non-ADHD peers [21,51,52].

Several factors have been proposed to explain why children with ADHD have lower rates of physical activity participation. Although children with ADHD often demonstrate excessive gross motor movement (hyperactivity), they are at greater risk for movement-related disorders such as developmental coordination disorder (DCD) [53], which reflects deficits in fundamental movement skills and slower progression in these skills compared to their non-ADHD peers [54]. Children with poor fundamental movement skills often display low confidence in their physical abilities [55], which may lead children with ADHD to avoid physical activities that could help them develop these skills [56]. As a result, children with ADHD may find themselves in a debilitating cycle of poor fundamental movement skills, impaired self-confidence, and physical activity avoidance, which perpetuates decreased participation [54].

In addition to a developmental lag in fundamental movement skills, children with ADHD may view the purpose of physical activity differently than their peers, leading to social exclusion from participation. Interestingly, children with ADHD often view the primary goal of physical activity as socialization rather than competition [54]. This lack of conceptual understanding regarding the purpose of physical activity may raise challenges with their peers such as misaligned goals, which may deter them from participation. The provision of clear instructions and definitions on sport and physical activity concepts and terms may promote a holistic understanding of fundamental concepts, effective communication, skill development, and greater social cohesion and inclusion in physical activity. Although children with ADHD who engage in more physical activity overall show greater symptom management, executive functioning [57], and mood [17], children with ADHD who participate in sports specifically have fewer internalizing symptoms of depression and anxiety [58,59]. It may be beneficial for children with ADHD to be consulted on both the social and competitive aspects of physical activity and sports, with the intention of helping align their expectations with those of their peers in order to foster improved social interactions in physical activity environments.

Another contributing factor to reduced physical activity participation among children with ADHD relates to disordered self-regulation, a hallmark characteristic of ADHD [60,61,62,63]. Self-regulation is essential for sustained participation in activities that require consistent effort, attention, and the maintenance of thoughts, emotions, and behaviours in the pursuit of goals. The deficits in self-regulation observed in children with ADHD may act as a barrier to physical activity engagement, as these activities require ongoing effort and self-monitoring. Self-regulation also involves the ability to perceive and respond to physiological processes [63], with prior work underscoring a strong association between emotion regulation and interoception—the awareness of internal bodily signals [60,63,64,65]. Children who can accurately perceive internal bodily signals are more likely to adopt effective regulatory strategies and adjust their behaviour accordingly depending on environmental cues [57,64]. Interoception deficits have been identified in several disorders, including autism spectrum disorder, tic disorder, anxiety, depression, as well as ADHD [65,66,67,68,69,70,71]. Higher levels of physical activity and physical fitness have been positively associated with better interoceptive awareness in primary school children [65,72]. Physical activity may serve as a form of interoceptive exposure, allowing individuals to become more familiar with their bodily signals through repeated experience [65,73]. This dynamic relationship suggests that interoceptive processes help regulate exertion during physical activity, while physical activity enhances the processing of internal bodily signals [65]. Taken together, deficits in psychological and physiological self-regulation mechanisms may contribute to lower physical activity engagement in children with ADHD compared to their peers. Counselling professionals and educators can help address these deficits by emphasizing self-regulatory techniques that children with ADHD can develop and apply during physical activity to enhance their participation. For example, mindfulness practices, yoga interventions [74,75,76] and integrative mind–body training (IBMT) [77,78] have been shown to improve executive functioning, behavioural regulation and metacognition in children with and without ADHD [79]. Importantly, these techniques aim to improve the mind–body connection through various practices targeting self-awareness and self-monitoring. Other techniques that promote self-awareness and self-monitoring skills include journaling, behaviour tracking, and goal setting [80,81]. These strategies may work to enhance self-regulation, self-monitoring, and interoceptive awareness in children with ADHD.

**Research Objective** **2:** **How do children perceive the benefits of physical activity based on their knowledge and personal experiences?**

Children with ADHD were less aware of the physical and mental health benefits of physical activity compared to children without ADHD, and they also reported athletic performance as a benefit of physical activity more frequently than their non-ADHD peers. These findings align with other work showing that children with ADHD often lack a conceptual understanding of the value of physical activity and have more difficulty relating to the competitive goals of sports [54]. Children with ADHD may perceive physical activity as a distinct or compartmentalized activity, restricted to social settings and primarily related to developing athletic performance skills, rather than an integral part of everyday life that supports overall health.

A greater proportion of males compared to females endorsed the theme of benefits to the body and benefits for athletic performance in both the ADHD and non-ADHD groups. These results may reflect the superficial knowledge of physical activity demonstrated in boys with ADHD, as outlined by Harvey et al. [34], in which they demonstrate performative tendencies with the intent to increase their own social desirability. Additionally, more males compared to females in both groups endorsed physical activity as an enriching activity, being fun and engaging. This may be the result of greater overall physical activity participation observed in males, which may facilitate more opportunities for them to have these enriching experiences. Furthermore, more females than males in both groups endorsed the benefits of physical activity for their mental health. This pattern of findings could reflect the higher prevalence of internalizing difficulties (e.g., anxiety, depression) among female compared to male children [82], combined with a potentially more developed introspective capacity of internal states among female children [83]. Thus, greater instances of internalizing difficulties and greater self-awareness may increase their sensitivity to the regulating effects of physical activity.

Taken all together, children with ADHD demonstrated less awareness of the physical and mental health benefits of physical activity compared to children without ADHD. Children with ADHD also reported athletic performance as a benefit of physical activity more frequently than their non-ADHD peers, potentially due to the socially facilitative effects of athletic ability. And, overall, females had a greater sense of the benefits of physical activity for mental health compared to males, potentially due to more experiences with internalizing difficulties coupled with more developed self-awareness.

**Research Objective** **3:** **How do children perceive the impact of physical activity on their mood and their ability to focus?**

Children with ADHD discussed the importance of physical activity for their mood more frequently compared to children without ADHD. Children with ADHD described experiencing a variety of positive feelings after engaging in physical activity, such as feeling happy, excited, relaxed, calm, stress-free, nicer, and rectifying feelings of upset or boredom. Physical activity has been shown to stimulate prefrontal cortical activity and upregulate dopamine release [84], which can promote a sense of calm while also reducing anxiety, depression, and mood symptoms in children with ADHD [84]. Thus, mood-related challenges experienced by children with ADHD have the potential to be alleviated through physical activity participation. In contrast, children without ADHD who endorsed physical activity improving their mood provided less varied and detailed responses. The pronounced impact of physical activity on the mood of children with ADHD may provide them with more opportunities to recognize and articulate their emotional experiences. This suggests that physical activity may enhance emotional awareness, expression, and interoception [65], particularly in children with ADHD. Research has suggested that interoception may mediate the benefits of physical activity for both emotional [73] and cognitive processes [65,85]. Several children without ADHD noted that their perception of how physical activity affects their mood depended on contextual factors, such as the level of fun, difficulty, variety, and intensity of the activity. These findings support previous work, underscoring the more consistently positive impact of physical activity on mood in children with ADHD [65].

Furthermore, children with ADHD more frequently reported improved focus following physical activity than children without ADHD. Some children with ADHD expressed that physical activity enhances their overall mental state (e.g., making them calm, clearing their mind, or providing a fresh start), which they felt subsequently helped them focus. This further highlights the role of physical activity in improving mental well-being and suggests that physical activity benefits for executive functioning may be mediated by improvements to mental well-being, which has received support in previous research [86,87]. In contrast, some children without ADHD reported that physical activity hindered their focus, attributing this to fatigue or loss of energy. These perceptions may stem from experiences with high-intensity, vigorous activity that naturally leads to tiredness or may reflect variations in fitness levels among participants. Educational settings should be mindful of these differences when incorporating physical activity into the classroom and offer a range of physical activity options.

In summary, children with ADHD viewed physical activity as more impactful on their mood and their ability to focus to a greater extent than children without ADHD. These findings may reflect a connection between engaging in physical activity and improvements in interoception (i.e., awareness of internal bodily signals), which children with ADHD may benefit the most from.

**Research Objective** **4:** **What are children’s views of using desk cycles during learning tasks and in classroom environments?**

Children with ADHD viewed the use of desk cycles in a classroom as helpful for their focus and learning to a greater extent than those without ADHD and expressed a significantly greater desire for the use of desk cycles in classroom spaces. These findings coalesce with other research also showing the benefits of increased gross motor movement (desk cycling, fidgeting) during executive functioning on performance outcomes and self-efficacy in children with ADHD [1,21,88]. Simultaneous physical activity may have a holistic, multilayered effect in children with ADHD, whereby various systems within self-regulation, including emotion regulation (i.e., awareness, expression), interoception, and executive functioning (i.e., inhibition, task completion/follow-through), influence and enhance one another.

However, using desk cycles and encouraging simultaneous physical activity and executive functioning are not a one-size-fits-all solution. Several children from both groups reported the desk cycle was distracting during executive functioning and would be similarly distracting during classroom learning. Some children, with and without ADHD, raised concerns around multitasking, suggesting that performing two tasks at once could lead to competing mental efforts. This concern coincides with previous research showing both children with and without ADHD have impaired performance when completing a dual-task paradigm requiring divided attention (i.e., multitasking) [89,90]. However, as noted earlier, other children preferred multitasking, especially those with ADHD who welcomed the ability to expend physical energy while focusing mental energy. Differences in fitness levels, overall executive functioning, and ADHD subtypes (i.e., inattentive, hyperactive/impulsive, combined) may play a role in the impact of multitasking with movement. Recent work shows that only those with the predominantly hyperactive ADHD subtype benefit from movement during learning, whereas those with the predominantly inattentive subtype benefit from remaining stationary during attention-demanding tasks [91]. More research is needed to explore multitasking with movement and why some individuals find it more helpful than others.

More males than females with ADHD endorsed the desk cycle’s utility during learning tasks and in classroom environments. These findings are consistent with previous work given that most males with ADHD have hyperactive/impulsive symptoms, and hyperactive symptoms benefit from simultaneous movement and learning [88,91]. In contrast, females are more often diagnosed with inattentive ADHD [40,92,93], rather than hyperactive, in which case simultaneous movement and executive functioning may be more distracting than helpful. These findings emphasize the importance of considering individual difference factors such as the ADHD subtype and gender when designing physical activity interventions to optimize outcomes across a diverse range of children.

**Limitations**

The current study used self-report data from children aged 8–12 years old, which carries certain limitations. One criticism is that children often have less robust introspection abilities or less verbally expressive capability, potentially limiting their ability to recognize and express their knowledge of and experiences with physical activity, as well as its impact on health and well-being. To address this limitation as best as possible, researchers simplified all language during interviews and used probing questions to help children more effectively reflect on their knowledge and experiences. Another limitation is that the current study did not consider how children’s physical fitness levels may moderate their experience with desk cycling and the effects of movement during executive functioning. Indeed, children with higher fitness levels enjoy physical activity more than children with lower fitness levels [94]. As a result, children with higher fitness levels may be more accustomed to novel physical activity experiences or be more familiar with the physiological arousal that accompanies physical activity engagement, leaving them more open to the desk cycle experience. Conversely, children with lower fitness levels may have a more negative perception of the desk cycle due to less experience with physical activity paradigms in general. Further, a larger and more representative sample with children from different schools and socio-economic backgrounds would help strengthen the conclusions of the current work. Integrating objective measures using accelerometers and cognitive tests would also be beneficial to provide a more comprehensive analysis of the effects of physical activity on ADHD symptoms and learning outcomes. Additionally, although the current study treated ADHD as a single condition, it would be valuable for future work to consider variance in ADHD symptomology by analysing ADHD subtypes (i.e., inattentive, hyperactive, combined) to offer insight into tailored physical activity recommendations for diverse ADHD needs. Finally, the limited number of female participants in both ADHD and non-ADHD groups hindered the robustness of statistical analyses on within-group gender differences. Consequently, the reported gender differences are based on the observed proportion of males and females endorsing each theme within their respective diagnostic groups. This male predominance is a recurring limitation in ADHD research [95]. More inclusive samples are needed in future research, allowing for the investigation of gender differences in ADHD, physical activity, and movement-based learning.

**Practical applications**

The current study further underscores the inequity in physical activity experiences between children with ADHD and their neurotypical peers. This work points parents, teachers, researchers, and the broader community to continue identifying ways to promote physical activity opportunities for neurodivergent children, who stand to reap immense physical, cognitive, and psycho-emotional benefits from regular physical activity engagement. Further, this research demonstrates that children with ADHD are aware of their physical activity needs above and beyond what their peers without ADHD require, especially as it relates to their mood and ability to focus. It is essential that educational environments, school classrooms, and home settings make available physical activity outlets for those children who stand to benefit from greater access to movement during the day. With that said, this work also delineates that one size does not fit all and that in classroom environments, children should be provided with a choice to engage in physical activity throughout the day, but that engagement should not necessarily be made compulsory during learning tasks, as that approach may not benefit all.

## 5. Conclusions

This study emphasizes key differences in the physical activity experiences and preferences between children with and without ADHD. Compared to children without ADHD, children with ADHD reported lower physical activity levels, more often emphasized the benefits of movement for improving mood and focus during learning, viewed classroom-based desk cycling as a helpful tool to focus their attention, and expressed a desire to use desk cycling during classroom learning. Critically, however, the use of desk cycling during classroom learning should not be a one-size-fits-all instructional approach, as many children viewed physical activity while learning, such as desk cycling during executive functioning, as multitasking and distracting. Classroom learning may be enhanced by offering optional physical activity outlets for those who identify as benefiting from movement during learning. Future work should consider evaluating the impact of movement options in children’s classrooms to determine the utility of movement for diverse children. While some previous work has examined structured physical activity breaks in classrooms, the idea is to provide movement options that children can opt into rather than movement obligations, in order to offer great inclusivity for diverse needs. A combination of teacher reports, student reports and objective metrics (accelerometers) both acutely and over the long term can be used to holistically capture the role of movement in classrooms.

## Figures and Tables

**Table 1:** Research and interview questions.

	Research Question	Interview Question(s)
1.	What physical activities do children with and without ADHD typically participate in?	What physical activities do you participate in?
2.	How do children with and without ADHD perceive the benefits of physical activity based on their (a) knowledge and (b) personal experience?	Do you know of any benefits of physical activity?
3.	How do children perceive the impact of physical activity on their mood and their ability to focus?	Does physical activity change your mood? Does your ability to focus change after physical activity?
4.	What are children’s views of using desk-cycles during learning tasks, and in classroom environments?	Did you find the desk cycling helpful or distracting? Would you use something like a desk- cycle in your classroom if it was available?

**Table 2:** Demographic information for participants with and without ADHD and their guardians.

Characteristic	ADHD	Non-ADHD
Age of Child (years), mean (SD)	9.83 (1.58)	10.043 (1.26)
Age of Guardian (years), mean (SD)	40.625 (7.69)	43.826 (4.76)
Sex of Participant		
Male	17 (71)	15 (65)
Female	7 (29)	8 (35)
Sex of Guardian		
Male	3 (12)	3 (13)
Female	21 (88)	20 (87)
Guardian’s Education Level (n)		
Some high school, no diploma	0 (0)	0 (0)
High school graduate, diploma or the equivalent	1 (4)	0 (0)
Some college credit, no degree	1 (4)	2 (9)
Trade/technical/vocational training	4 (17)	1 (4)
Associate degree	2 (8)	1 (4)
Bachelor’s degree	7 (29)	7 (30)
Master’s degree	5 (21)	8 (35)
Professional degree	3 (13)	4 (17)
No response	1 (4)	0 (0)
Guardian’s Employment (n)		
Employed for wages	16 (67)	11 (48)
Self-employed	4 (17)	6 (26)
Out of work	2 (8)	3 (13)
Homemaker	1 (4)	3 (13)
Student	1 (4)	0 (0)
No response	0 (0)	0 (0)
Household Income		
Prefer not to say	2 (8)	5 (22)
<$30,000	1 (4)	2 (9)
$30,000–$40,000	0 (0)	1 (4)
$40,000–$50,000	1 (4)	1 (4)
$50,000–$60,000	2 (8)	4 (17)
$60,000–$70,000	2 (8)	2 (9)
$70,000–$80,000	0 (0)	1 (4)
$80,000–$90,000	2 (8)	1 (4)
$90,000–$100,000	3 (13)	0 (0)
>$100,000	11 (46)	6 (26)
Age of ADHD diagnosis (n)		N/A
Unsure	2 (8)	--
3–5	4 (17)	--
6–8	13 (54)	--
9–12	5 (21)	--
Currently Taking ADHD Medication		--
No response	2 (8)	--
Yes	14 (58)	--
No	8 (33)	--
Other Diagnosis Present		--
Yes	7 (29)	--
No	17 (71)	--
Medicated for Another Diagnosis		--
Yes	8 (33)	--
No	16 (67)	--
No Response	0 (0)	--

**Table 3:** Frequency summary and chi-squared comparisons of main themes related to physical activities in which children with and without ADHD participate.

Theme	ADHD Frequency: 73	ADHD Male (M) vs. Female (F)	Theme	Non-ADHD Frequency: 91	Non-ADHD Male (M) vs. Female (F)
**Individual** activities *	**24**	M = 10/15 (67%) F = 5/9 (56%)	**Individual** activities *	**34**	M = 9/16 (56%) F = 6/7 (86%)
Team sports *	**24**	M = 10/15 (67%) F = 4/9 (44%)	Team sports *	**34**	M = 11/16 (69%) F = 6/7 (86%)
Non-structured activities	10	M = 4/15 (27%) F = 3/9 (33%)	School-based activities	10	M = 3/16 (19%) F = 3/7 (43%)
Disinterest in physical activity	8	M = 4/15 (27%) F = 3/9 (33%)	Disinterest in physical activity	7	M = 4/16 (25%) F = 2/7 (29%)
School-based activities	7	M = 2/15 (13%) F = 3/9 (33%)	Non-structured activities	6	M = 4/16 (25%) F = 2/7 (29%)

Note. * **Individual Activities**, chi-squared test χ^2^ (2, 42) = 5.81, *p* = 0.05; * Team Sports, chi-squared test χ^2^ (2, 42) = 5.81, *p* = 0.05 *; Non-Structured Activities chi-squared test χ^2^ (1, 42) = 1.62, *p* = 0.20; Disinterest in Physical Activity chi-squared test χ^2^ (1, 42) = 0.10, *p* = 0.75; School-Based Activities, chi-squared test χ^2^ (1, 42) = 0.89, *p* = 0.35.

**Table 4:** Frequency summary and chi-squared comparisons of main themes related to benefits of physical activities based on participants’ knowledge.

Theme	ADHD Frequency: 54	ADHD Male (M) vs. Female (F)	Theme	Non-ADHD Frequency: 46	Non-ADHD Male (M) vs. Female (F)
Body	22	M = 11/15 (73%) F = 5/9 (56%)	Body	22	M = 8/16 (50%) F = 3/7 (43%)
Unsure *	**9**	M = 5/15 (33%) F = 3/9 (33%)	Overall health *	**16**	M = 11/16 (69%) F = 2/7 (29%)
Overall health *	**8**	M = 3/15 (20%) F = 3/9 (33%)	Mental processes	4	M = 2/16 (13%) F = 1/7 (14%)
**Athletic** performance *	**8**	M = 4/15 (27%) F = 1/9 (11%)	Unsure *	**3**	M = 1/16 (6%) F = 2/7 (29%)
Mental processes	7	M = 3/15 (20%) F = 3/9 (33%)	**Athletic** performance *	**1**	M = 1/16 (6%) F = 0/7 (0%)

Note. Body, chi-squared test χ^2^ (1, 42) = 0.00, *p* = 1.00; * Unsure, chi-squared test χ^2^ (1, 42) = 4.20, *p* = 0.04 *; * Overall health, chi-squared test χ^2^ (1, 42) = 6.22, *p* = 0.013; * Athletic performance, chi-squared test χ^2^ (1, 42) = 6.93, *p* = 0.008 *; mental processes, chi-squared test χ^2^ (1, 42) = 1.109, *p* = 0.292.

**Table 5:** Frequency summary and chi-squared comparisons of main themes related to benefits of physical activity based on participants’ personal experience.

Theme	ADHD Frequency: 21	ADHD Male (M) vs. Female (F)	Theme	Non-ADHD Frequency: 19	Non-ADHD Male (M) vs. Female (F)
Enriching activity	12	M = 8/15 (53%) F = 1/9 (11%)	Enriching activity	11	M = 6/16 (38%) F = 1/7 (14%)
Personal growth	5	M = 1/15 (7%) F = 2/9 (22%)	Mental state	5	M = 1/16 (6%) F = 2/7 (29%)
Mental state	4	M = 1/15 (7%) F = 1/9 (11%)	Personal growth	3	M = 2/16 (13%) F = 0/7 (0%)

Note. Enriching activity, chi-squared test χ^2^ (1, 42) = 0.096, *p* = 0.757; personal growth, chi-squared test χ^2^ (1, 42) = 0.618, *p* = 0.432; mental state, chi-squared test χ^2^ (1, 42) = 0.141, *p* = 0.707.

**Table 6:** Frequency summary and chi-squared comparisons of main themes related to participants’ perceived effects of physical activity on their ability to focus.

Theme	ADHD Frequency: 45	ADHD Male (M) vs. Female (F)	Non-ADHD Frequency: 45	Non-ADHD Male (M) vs. Female (F)
Physical activity helps focus	25	M = 7/15 (47%) F = 8/9 (89%)	22	M = 10/16 (63%) F = 6/7 (86%)
Mixed perception of physical activity effects on focus	9	M = 5/15 (33%) F = 3/9 (33%)	10	M = 4/16 (25%) F = 4/7 (57%)
Physical activity hinders focus	7	M = 2/15 (13%) F = 2/9 (22%)	8	M = 4/16 (25%) F = 2/7 (29%)
Physical activity does not change focus	4	M = 2/15 (13%) F = 2/9 (22%)	5	M = 5/16 (31%) F = 0/7 (0%)

Note. Physical activity helps focus, chi-squared test χ^2^ (2, 42) = 0.326, *p* = 0.220; mixed perception, chi-squared test χ^2^ (1, 42) = 0.096, *p* = 0.757; physical activity hinders focus, chi-squared test χ^2^ (1, 42) = 0.104, *p* = 0.747; physical activity does not change focus, chi-squared test χ^2^ (1, 42) = 0.141, *p* = 0.707.

**Table 7:** Frequency summary and chi-squared comparisons of main themes related to participants’ perceived effects of physical activity on their mood.

Theme	ADHD Frequency: 50	ADHD Male (M) vs. Female (F)	Theme	Non-ADHD Frequency: 51	Non-ADHD Male (M) vs. Female (F)
**Physical** activity helps mood *	32	M = 12/15 (80%) F = 6/9 (67%)	**Physical** activity helps mood *	**26**	M = 11/16 (69%) F = 5/7 (71%)
Mixed perception of effects of physical activity on mood	9	M = 3/15 (20%) F = 4/9 (44%)	Physical activity hinders mood	11	M = 5/16 (31%) F = 4/7 (57%)
Physical activity hinders mood	5	M = 2/15 (13%) F = 2/9 (22%)	Mixed perception of effects of physical activity on mood	9	M = 5/16 (31%) F = 2/7 (29%)
Physical activity does not change mood	4	M = 1/15 (7%) F = 2/9 (22%)	Physical activity does not change mood	5	M = 4/16 (25%) F = 0/7 (0%)

Note.* **Physical activity helps mood**, chi-squared test χ^2^ (2, 42) = 12.62, *p* = 0.002; mixed perception, chi-squared test χ^2^ (1, 42) = 0.382, *p* = 0.537; physical activity hinders mood, chi-squared test, χ^2^ (1, 42) = 1.714, *p* = 0.190; physical activity does not change mood, chi-squared test χ^2^ (1, 42) = 0.141, *p* = 0.707.

**Table 8:** Frequency summary and chi-squared comparisons of main themes related to participants’ views of using the desk cycle during learning tasks.

Theme	ADHD Frequency: 47	ADHD Male (M) vs. Female (F)	Non-ADHD Frequency: 41	Non-ADHD Male (M) vs. Female (F)
**Desk-cycle was** helpful *	**28**	M = 10/15 (67%) F = 5/9 (56%)	**22**	M = 11/16 (69%) F = 5/7 (71%)
Desk-cycle was distracting	12	M = 4/15 (27%) F = 5/9 (56%)	10	M = 5/16 (31%) F = 2/7 (29%)
Mixed perception of desk-cycle utility	7	M = 2/15 (13%) F = 4/9 (44%)	9	M = 3/16 (19%) F = 3/7 (43%)

Note.* **Desk cycle was helpful**, chi-squared test χ^2^ (1, 42) = 5.559, *p* = 0.018; desk cycle was distracting, chi-squared test χ^2^ (1, 42) = 0.382, *p* = 0.537; mixed perception, chi-squared test χ^2^ (1, 42) = 0.404, *p* = 0.525.

**Table 9:** Frequency summary and chi-squared comparisons of main themes related to participants’ views of using the desk cycle in classroom learning environments.

Theme	ADHD Frequency: 52	ADHD Male (M) vs. Female (F)	Non-ADHD Frequency: 47	Non-ADHD Male (M) vs. Female (F)
**Desire to use** desk-cycle *	**30**	M = 13/16 (81%) F = 6/9 (67%)	**24**	M = 10/16 (63%) F = 5/7 (71%)
Disinterest in using desk-cycle	11	M = 4/16 (25%) F = 3/7 (43%)	13	M = 2/7 (30%) F = 6/16 (38%)
Mixed perception of usefulness of desk-cycle	11	M = 5/15 (33%) F = 2/9 (22%)	10	M = 6/16 (38%) F = 3/7 (43%)

Note.* **Desire to use desk-cycle**, chi-squared test χ^2^ (1, 42) = 4.2, *p* = 0.040; disinterest in using desk cycle, chi-squared test, χ^2^ (1, 42) = 0.389, *p* = 0.533; mixed perception, chi-squared test χ^2^ (1, 42) = 0.095, *p* = 0.758.

## Data Availability

The data presented in this study are available upon request from the corresponding author. The data are not publicly available due to ethical restrictions.

## Supplementary Materials

The following supporting information can be downloaded at: https://www.mdpi.com/article/10.3390/sports13080240/s1.
