# Supplementary material for: Perspectives on Physical Activity and Learning from Children With and Without ADHD
Source: Sports (Basel). 2025 Jul 22;13(8):240. doi: 10.3390/sports13080240 (PMC12390025; doi:10.3390/sports13080240)
Supplement: Supplementary file 1 [file sports-13-00240-s001.zip › sports-3767237-supplementary.pdf]

The following provides representative quotations by participants for each research question.

Research Question 1: What physical activities do children with and without ADHD typically participate in?

Theme 1: Individual activities (ADHD frequency 24; non-ADHD frequency 34). Participants with and without ADHD expressed that individual activities were the most common physical activities in which they engage. Some participants mentioned, "I like swimming and running" (Participant 8, female, ADHD); and "I like rock climbing" (Participant 36, male, non-ADHD). However, children with ADHD expressed significantly less involvement with individual activities compared to those without ADHD ($p=.05$).

Theme 2: Team sports (ADHD frequency 24; non-ADHD frequency 34). Participants with and without ADHD discussed various team sports they participate in, including hockey, ringette, baseball, softball, soccer, and football. Children with ADHD expressed significantly less involvement with team sports compared to those without ADHD ($p=.05$).

Theme 3: Non-structured activities (ADHD frequency 10; non-ADHD frequency: 6). Participants with and without ADHD noted a variety of non-structured activities they participate in, including building snowballs, playing tag, tobogganing, strategy games, and relation video games (physical video games).

Theme 4: Disinterest in physical activity (ADHD frequency 8; non-ADHD frequency: 7). Both groups endorsed this theme the fourth most frequently and expressed their disinterest with physical activity as a whole, with certain physical activities, and/or their preference for sedentary activities. Some participants expressed that they do not enjoy running laps in gym class and would rather play video games or relax. The overall sentiment can be captured by quotes such as "I don't love sports" (Participant 16, male, non-ADHD), and "I do not enjoy physical activity" (Participant 23, male, ADHD).

Theme 5: School-based activities (ADHD frequency: 7; non-ADHD frequency 10). All participants shared a variety of school-based activities they participate in during physical education class or recess. Some activities included floor is lava, tag, manhunt, foursquare, physical education class, gym, and soccer. School-based activities were the most infrequently endorsed form of physical activity in the ADHD group whereas, it was the third most popular theme in the non-ADHD group.

Research Question 2: How do children with and without ADHD perceive the benefits of physical activity based on their (1) knowledge and (2) personal experience?

Perceptions of Physical Activity Benefits Based on Personal Knowledge

Theme 1: Benefits for the body (ADHD frequency 22; non-ADHD frequency 22). Participants described physical activity as positively affecting the body, and this theme was the most common in both groups. Participants with ADHD mentioned "You become stronger" (Participant 13, female); "Physical activity helps you move better" (Participant 29, male); and "Physical activity improves your arm strength and your leg strength" (Participant 35, female). Participants without ADHD mentioned "[Physical activity] gets your blood circulating" (Participant 40, male); "Physical activity gets your endurance higher so you can run farther, you can run faster, and it strengthens your muscles" (Participant 10, male); "You're stronger…like your muscles and bones get stronger" (Participant 15, male); and "Physical activity makes

you healthier and stronger. You get better sleep. I think it just makes you stronger and nicer. And you don't sit down all the time" (Participant 33, female).

Theme 2: Unsure of benefits (ADHD frequency 9; non-ADHD frequency 3). Many participants stated that they were unaware of the benefits of physical activity: "No [I don't know the benefits of physical activity]" (Participant 27, male; Participant 28, female). Chi-squared tests revealed that those with ADHD were significantly more unsure of the benefits of physical activity compared to those without ADHD (p=.04).

Theme 3: Benefits for overall health (ADHD frequency 8; non-ADHD frequency 16). Participants described physical activity as a broadly health-promoting behaviour. Children without ADHD discussed the benefits of physical activity for overall health significantly more than those with ADHD (p=.013). Several participants with ADHD described physical activity as "…helping you stay active" (Participant 9, female); "…helping you to be healthier, which can help you live longer" (Participant 14, female; participant 44 male); and "helping you to not become a couch potato" (Participant 41, female). Several participants without ADHD mentioned "There are benefits of just moving. Like not sitting all day is just better because it's like oil for your gears" (Participant 18, male); "Walking gives you fresh air" (Participant 6, male); and "If you're sick, sometimes you need to exercise and it'll make you feel better" (Participant 8, female).

Theme 4: Athletic performance benefits (ADHD frequency 8; non-ADHD frequency 1). Participants described physical activity as helping to improve athletic performance. Chi-squared analysis confirmed that children with ADHD discussed the benefit of physical activity for athletic performance significantly more than those without ADHD (p=.008). Participants without ADHD described athletic performance benefits from physical activity as "…a competitive thing. You can be competitive, and you can get on top" (Participant 41, female); "It makes your brain be able to send signals…like experience…if you did hockey a whole bunch, you'd be able to do it quicker" (Participant 29, male); and "it can help you know how to play the game…you know the rules of the game better" (Participant 31, male).

Theme 5: Mental processes (ADHD frequency 7; non-ADHD frequency 4). Participants described physical activity as a helpful way to enhance both the brain's physical structures and processes, as well as its mental functioning. Several participants with ADHD described physical activity supporting mental processes by "helping the brain react faster to things" (Participant 9, female), and "helping with mental health" (Participant 32, female). Participants without ADHD stated physical activity "benefits brain health…and helps brain activity" (Participant 12, female), and "stimulates your brain functions" (Participant 19, male).

Perceptions of Physical Activity Benefits Based on Personal Experience

Theme 1: Enriching activity (ADHD frequency 12; non-ADHD frequency 11). Participants described their personal experience with physical activity as being fun and engaging, and this was the most frequently endorsed theme in both groups. Several participants with ADHD said "I have fun" (Participant 17, male), "It gets me active" (Participant 39, male), and "I enjoy it because there's a lot of engagement and there's a wide variety of it" (Participant 42, male). Participants without ADHD similarly said "[I like] trail running because it's outdoors. And the terrain is fun to explore" (Participant 15, male), "It's just generally fun" (Participant 16, male), and "I like physical activity because it lets me have fun" (Participant 21, male).

Theme 2: Personal growth (ADHD frequency 5; non-ADHD frequency 3). Participants described physical activity as helping them feel more accepted in their community, as providing an opportunity to explore

new activities and skills, and as eliciting feelings of accomplishment for doing challenging but rewarding activities. Several participants with ADHD mentioned "I'm not very accepted in the community, so I think [doing physical activity] might be a good way to get accepted" (Participant 32, female); "[Physical activity] gives me more new things to try" (Participant 30, female); and "It feels good to accomplish things and engage in activities that are important, like helping my dad with work and with heavy lifting" (Participant 42, male). One participant without ADHD described developing new skills through physical activity: "When I first started karate, I didn't know much, but I learned more and became better at it" (Participant 38, male) which ultimately fueled further interest and engagement.

Theme 3: Mental state (ADHD frequency 4; non-ADHD frequency 5). Participants described physical activity as improving their mental state. Participants with ADHD mentioned "[Physical activity] makes me more of a carefree person, more stress free" (Participant 32, female); "I stop thinking about bad things [when I'm physically active]" (Participant 39, male); and "[Physical activity] gets your mind off things and makes your mind more positive" (Participant 39, male). Participants without ADHD noted "You don't really think about the physical activity. You're not really thinking about anything, and then you just become happier" (Participant 18, male); and "I can get my energy out and that feels nice" (Participant 43, female).

Research Question 3: How do children perceive the impact of physical activity on their (1) focus and (2) mood?

The Effects of Physical Activity on Focus

Theme 1: Physical activity helps focus (ADHD frequency 25; non-ADHD frequency 22). Physical activity was most frequently described as helping promote focus in both groups. A few statements made by participants with ADHD indicated that physical activity helped their focus due to a change in their mental state, influencing quality of mind, calmness, or a 'fresh start' mindset. Participants with ADHD expressed, "I usually don't focus [well] in the morning, but after Physical Education class, I start focusing a lot better" (Participant 13, female), "[Being physically active] feels like a fresh start, and if I was frustrated, taking a [physical activity] break helps me come back with a better mindset" (Participant 42, male), and "[Physical activity] helps me focus more on writing" (Participant 30, female). Noteworthy statements made by a few participants without ADHD indicated that physical activity promoted their focus by helping them release excess energy, with several noting: "It's easier to concentrate [after physical activity]" (Participant 10, male), "Usually, I feel like I have a lot of energy that needs to be spent, but after I do physical activity, all that energy is spent, so I can focus" (Participant 16, male), "That's why I like recess, because after we're running around and everything, I can just come inside and focus better" (Participant 18, male), and "If it gets my energy out, I'm calmer" (Participant 44, female).

Theme 2: Mixed perception of physical activity effects on focus (ADHD frequency 9; non-ADHD frequency 10). Participants in both groups expressed being unsure of the effects of physical activity on their focus, with some also noting that their focus depends on situational factors. Some ADHD participants reported that it was hard to tell whether physical activity affects their focus, and other participants reported that sometimes physical activity improves their focus and sometimes it does not. The following statement was made by one participant with ADHD:
"If I don't have that much energy, I'm able to focus. But if I have too little energy, I can't focus, and I just want to do nothing. For example, if I had to do something simple but it required a lot of energy over a long period, I wouldn't be able to focus or complete it." (Participant 14, female).

Similarly, participants without ADHD reported that occasionally physical activity helps their focus and occasionally it does not. One participant indicated that situational factors are more likely to influence their focus, "If it's a lighter [physical] activity, then it helps me wake up a bit. But if I am just exhausted, I don't think I can do it too much and it doesn't help." (Participant 33, female).

Theme 3: Physical activity hinders focus (ADHD frequency 7; non-ADHD frequency 8). Physical activity hindering focus was the third most frequently endorsed theme among both groups. One participant noted "Physical activity makes me less focused. Maybe because I [use up my energy doing physical activity], my brain is more tired than usual" (Participant 36, male). Several participants without ADHD indicated that physical activity typically hindered their focus due to a loss of energy, "Sometimes I'm just hot and sweaty and just want to lay down" (Participant 12, female); "I'm way too tired to focus [after]" (Participant 15, male); and "If I do too much, then I'm too tired to focus" (Participant 22, male).

Theme 4: Physical activity does not change focus (ADHD frequency 4; non-ADHD frequency 5). The least endorsed theme in both groups related to physical activity unaffecting participants' focus with one participant noting "No, I would never be able to focus on homework, [no matter what], I just don't like homework" (Participant 32, female). Participants without ADHD noted "Sometimes I'll be less silly but sometimes I'll not focus regardless [after physical activity]" (Participant 19, male); and "I think I'm the same [regardless of physical activity]" (Participant 20 and 26, both male).

The Effects of Physical Activity on Mood

Theme 1: Physical activity helps mood (ADHD frequency 32; non-ADHD frequency 26). The most frequently endorsed theme among both groups was physical activity helps mood. However, those with ADHD discussed the positive impact of physical activity on mood to a significantly greater extent than those without ADHD ($p=.002$). Participants with ADHD shared a variety of feelings experienced after engaging in physical activity including "more relaxed" (Participant 24, male), "happy and excited" (Participant 27 male), "more calm" (Participant 28, female), "Usually it's fun so I like doing it, so it makes me happy" (Participant 31, male), "Normally when I do physical activity when I'm not happy, it makes me happy after I do it" (Participant 35, female), "After going for a run, walk or bike ride, I feel more relaxed because I'm tired out" (Participant 41, female), and "When I'm tired, I have Physical Education in the morning as my second subject and it puts me in a good mood, not tired, but energized" (Participant 47, male). Several participants without ADHD expressed "Physical activity makes me more happy" (Participant 6, male), "When I just stay inside and play video games all day, I'm meaner, but if do physical activity, I'm nicer" (Participant 10, male), "When I do drama and when I dance, it makes me happier and I want to do it more" (Participant 12, female), "Physical activity makes me more excited and more happy and more relaxed" (Participant 25, male), and "If I'm bored, then I got exercise, and I feel better" (Participant 48, female).

Theme 2: Mixed views of the effect of physical activity on mood (ADHD frequency 9; non-ADHD frequency 9). Participants expressed that they were unsure of the effects of physical activity on their mood. Several participants with ADHD stated "It depends on how fun it is. If it's like basketball which I hate, I'll be like ughhh but if it's like dodgeball, I'll be like yayyy" (Participant 44, male); and "Sometimes [physical activity] gives me anger, sometimes happiness, sometimes sadness" (Participant 46, male). A few non-ADHD participants stated that their mood after physical activity is dependent on the context, including the level of fun, variety, difficulty, and intensity of the physical activity. Some comments included "Sometimes physical activity makes me happier and sometimes it makes me angrier" (Participant 8, female); "Sometimes when I have fun, physical activity helps my mood, but when I'm just

in a [bad] mood, sometimes I don't want to change my mood and then I'm grumpy all day" (Participant 15, male); "If it's fun physical activity, it makes me happy and I have fun. If it's annoying physical activity, like jumping jacks over and over, then I'm annoyed" (Participant 19, male); "Sometimes physical activity changes my mood. Sometimes it makes me a bit happier. Sometimes when I go too hard, it makes me a bit grumpy and I get mean" (Participant 33, female); and "I don't know how it makes me feel" (Participant 43, female).

Theme 3: Physical activity hinders mood (ADHD frequency 5; non-ADHD frequency 11). Although participants without ADHD most frequently described physical activity as benefitting their mood, the second most common theme was the opposite; physical activity was noted as hindering their mood. One participant with ADHD stated "Physical activity makes me less happy" (Participant 2, female); and another noted "If I have to do physical activity in the morning, it puts me in a bad mood" (Participant 13, female). More non-ADHD participants expressed a variety of negative feelings related to their mood after physical activity (e.g., tired, grumpy, angry, annoyed, mean).

Theme 4: Physical activity does not change mood (ADHD frequency 4; non-ADHD frequency 5). Also mentioned in both groups, although to lesser degrees, was that physical activity had no effect on mood.

Research Question 4: What are children's opinions on using desk-cycles during a) learning tasks, and b) in classroom environments?

Use of the Desk-Cycle During Learning Tasks

Theme 1: Desk-cycle was helpful during learning tasks (ADHD frequency: 28, non-ADHD frequency: 22). In both ADHD and non-ADHD groups, most participants expressed that using a desk-cycle during learning tasks can be helpful. Nonetheless, those with ADHD discussed the utility of desk-cycles to a greater extent than those without ADHD ($p=.018$). Some participants with ADHD expressed that using the desk cycle was helpful as the simultaneously movement facilitated their mental focus. Some participants also perceived their performance on the attention task was better while using the desk-cycle. Participants with ADHD stated, "It gives my legs something to do while I'm doing a task." (Participant 29, male); "It was helpful because it's a steady motion that's [easy to do]" (Participant 32, female); "The desk cycle was helpful… because I'm moving my body, and I don't like sitting still" (Participant 40, male); "I found it helpful; it allowed me to focus on the screen and release unwanted energy" (Participant 42, male); "It was very much helpful...I like to multitask a bunch of stuff, and this is basically multitasking" (Participant 41, female); and "It was helpful actually. I noticed that I actually did better with the bike…and my brain [was able to] memorize the keys" (Participant 44, male). Participants without ADHD provided similar comments such as "It somehow got me to concentrate a bit better" (Participant 10, male); "It was helpful. It was like when I come back from playing soccer, I get really focused in science" (Participant 34, female).

Theme 2: Desk-cycle was distracting during learning tasks (ADHD frequency: 12, non-ADHD frequency: 10). Using the desk-cycle during learning tasks was also described as distracting, and this was the second most common theme in both groups. Some participants with ADHD expressed that the desk-cycle was distracting during learning tasks due to the multitasking nature of engaging in both activities. Some mentioned "I had to concentrate more because part of my brain was thinking 'I must pedal, keep pedaling'. I had to focus lots of my energy on pedaling" (Participant 14, female); "[It was distracting because] I had to be doing two things at the same time" (Participant 24, male). In the non-ADHD group, several participants stated, "The desk cycling was a bit distracting because I had to do two things at the same time... I felt like I was running with a moving table" (Participant 38, male); "I thought it was

distracting... I had to focus on my pedaling and to make sure I didn't stop pedaling" (Participant 25, male).

<u>Theme 3:</u> Mixed views of desk-cycle utility (ADHD frequency: 7, non-ADHD frequency: 9). This was the most infrequently expressed theme in both the ADHD and non-ADHD group, and it indicates participants perceived the desk-cycle during learning tasks to be neutral. Participants in the ADHD group expressed, " I don't think it was good or bad...but it was pleasant" (Participant 36, male); "It was kind of both [positive and negative] at the same time" (Participant 31, male); "It was both helpful and not helpful because at first, I did better without the bike pedaling. But after a while, it actually increased my focus. It made me have less fails" (Participant 32, female). Several participants without ADHD expressed, "It was helpful, but sometimes it kept slowing me down" (Participant 8, female); "It didn't really do anything" (Participant 22, male); "It was sometimes a little hard to focus on the pedaling and the [task], but most of the time it was not that hard" (Participant 33, female).

<u>*Use of the Desk-Cycle in Classroom Learning Environments*</u>

<u>Theme 1:</u> Desire to use desk-cycles in classroom learning environments (ADHD frequency: 30, non-ADHD frequency: 24). The ADHD and non-ADHD groups both indicated a desire to use the desk-cycle in classroom learning environments. However, children with ADHD expressed a significantly greater desire to use desk-cycles compared to those without ADHD ($p=.040$). A few participants with ADHD described that they would use a desk-cycle in a classroom setting to cope with their general desire to move and feel motion, to release energy, and to combat boredom. Several indicated, "If I'm getting bored and just start fidgeting around, it could be helpful" (Participant 9, female); "It's fun to use and I like the motion while doing it" (Participant 32, female); "[Almost] everybody in class has a lot of trouble focusing. And so this could help us focus a lot." (Participant 30, female); "It would be beneficial to have some available in the back of the classroom for students who need a break or to expel energy" (Participant 42, male); "If I could use a desk-cycle, I would, because I'm always fidgeting... I don't really like fidgeting, biking is better" (Participant 44, male). Some participants without ADHD mentioned that using the desk-cycle during classroom instruction could help improve focus, alertness, and calmness. Several mentioned, "I actually listen more when I do this" (Participant 7, male); "It would make me concentrate" (Participant 10, male); "When I exercise, it calms me down and if I had a desk-cycle in a classroom, I would probably focus, and it would help" (Participant 12, female); "It would be helpful because it gives you energy to think about stuff" (Participant 45, male).

<u>Theme 2</u>: Disinterest in using desk-cycles in classroom learning environments (ADHD frequency: 11, non-ADHD frequency: 13). The ADHD and non-ADHD group both indicated a disinterest in using the desk-cycle in classroom learning environments. A few of the participants with ADHD noted concerns with using the desk-cycle due to potential social implications, including getting distracted by friends or distracting friends themselves. Several described, "I think it would be more distracting in a classroom, because [there are] more people" (Participant 29, male); and "If I had my friend during my class, I'd distract her" (Participant 35, female). A few participants without ADHD also reported that they would not use the desk-cycle in the classroom, as it would be challenging to multitask and concentrate on learning and completing schoolwork. Some participants noted, "It's a bit too distracting. It also takes away my energy" (Participant 15, male); I would prefer just sitting… If I'm biking in the middle of class, I wouldn't really be learning about math; I'd be more focused on biking than on the math" (Participant 43, female); and "I would not use the desk cycle…imagine trying to do a math question like 250 divided by ten while pedaling" (Participant 23, male).

Theme 3: Mixed views of desk-cycle utility in classroom learning environments (ADHD frequency: 11, non-ADHD frequency: 10). Participants in both groups reported a more neutral opinion regarding using the desk-cycle in classroom settings, indicating that they may have the desire to use the desk-cycle depending on situational factors. Participants with ADHD noted, "I think it would be good for some people. I learn better when I'm occupied with something in my hands [not my feet], but I do know someone who would benefit from this" (Participant 14, female); and "If I was doing homework, I would enjoy desk cycling more. Because this task had a time limit, the multitasking got tricky. Whereas if you were just doing homework and it wasn't timed, then it wouldn't be distracting but helpful" (Participant 24, male). Participants without ADHD commented, "Desk cycling can be helpful… except for the noise" (Participant 18, male); "If I have to focus really hard on something, the desk-cycle wouldn't be helpful. But if I'm working on something easier, it would be ok" (Participant 33, female).
